# Professor Dr. Med. Ludw. Hollænder

**Published:** 1897-07

**Authors:** 


### PROFESSOR DR. MED. LUDW. HOLLÆNDER.

PROFESSOR HOLLÆNDER, one of the most distinguished of the German dental profession, died March 12, 1897, from arterial calcification and degeneration of the muscles of the heart.

Dr. Hollænder was born in 1833, and, after passing through the Gymnasium, began the study of medicine in Breslau, Würzburg, and in Berlin. After graduation he practised medicine for seven years in South Africa, and subsequently continued in the same line of professional work in Berlin.

In 1871 he began the practice of dentistry and continued in this up to his death.

Dr. Hollænder was the translator and author of several works, and in addition was a voluminous contributor to periodical literature. This gave him a wide reputation extending far beyond the limits of his own country. His death will leave a vacancy in dental circles in Germany difficult to fill.

